# Megalourethra and urethrorectal fistula: a rare presentation and a challenging reconstruction

**DOI:** 10.1590/S1677-5538.IBJU.2015.0676

**Published:** 2017

**Authors:** Antonio Macedo, Sérgio Leite Ottoni, João Luiz Gomes Parizi, Gustavo Marconi Caetano Martins, Gilmar Garrone, Marcela Leal da Cruz

**Affiliations:** 1 Universidade Federal de São Paulo, SP, Brasil

## INTRODUCTION

Congenital megalourethra is a rare genital anomaly characterized by dilatation of the penile urethra with or without evidence of proximal or distal urethral obstruction. The urethra shows lack of corpus spongiosum and in some cases corpora cavernosa in the region of the distal urethra. The absence of these structures causes a ballooning of the urethra despite no mechanical obstruction. Some authors have also reported cases with prune-belly syndrome–like features (1, 2), so as the presence of urethral duplication (3).

We want to present a patient treated in our institution with megalourethra and urethrorectal fistula.

## MATERIAL AND METHODS

An 8-month male patient presented to our institution with history of anal micturition and an enhanced flaccid penis lacking corporal tissue. Physical examination showed a megalourethra and a rectal urethra at the anal border. The VCUG combined to retrograde urethrogram showed a normal bladder, a rectal urethra and a ballooned penile urethra, which ended blinded at the bulbar area without communication to the proximal segment. No previous history of UTIs and renal damage was found. We performed an ASTRA approach and isolated the rectal urethra, creating a perineal stump. We reconstructed the anal canal over the external sphincter. We then assessed the penile urethra by a longitudinal ventral incision enabling complete exposition of the dilated urethra. We dissected the distal penile urethra, which was opened and aligned to the perineal urethral stump by means of a termino-terminal anastomosis. We tailored the penile urethra over a 10F silicone tube and excised the redundant tissue. Finally, the penile skin was readapted after discarding the redundant skin. An indwelling tube was left for 10 days. Patient had a satisfactory outcome and excellent cosmetic result.

## DISCUSSION

Megalourethra is a rare malformation. Absence of the corpora cavernosa explains the massive dilatation of penile urethra despite mechanical obstruction. Congenital megalourethra has been classified into scaphoid and fusiform types and is usually associated with additional urinary tract and other system anomalies, irrespective of its type and severity. Amsalem et al. (4) reported on ten fetuses with megalourethra that were identified at a median gestational age of 19 (range, 13-24) weeks and all were confirmed postnatally or at autopsy. Three pregnancies were terminated and seven continued. All cases presented with a distended bladder and megalourethra and all cases had normal karyotype. Of seven liveborn babies, one died in the neonatal period due to pulmonary hypoplasia. All six infants alive had a dysfunctional urethra and three suffered from impaired or end-stage renal disease. Associated anomalies were found in half of the cases.

Operative technique for megalourethra with genital malformation has to be tailored to each individual case, depending on the intraoperative and endoscopic findings.

## CONCLUSION

Congenital megalourethra is caused by abnormal development or hypoplasia of the penile erectile tissue. When the amniotic fluid volume is normal, survival is possible but sexual dysfunction is expected. Urethroplasty follows the same principles of hypospadia repair.

## ARTICLE INFO

Available at: http://www.intbrazjurol.com.br/video-section/macedo_172_173/

Int Braz J Urol. 2017; 43 (Video #4): 172-4
